# Correlates of Sexually Transmitted Infections among Adolescents Attending Public High Schools, Panama, 2015

**DOI:** 10.1371/journal.pone.0163391

**Published:** 2016-09-22

**Authors:** Amanda Gabster, Debbie Y. Mohammed, Griselda B. Arteaga, Omar Castillero, Nataly Mojica, José Dyamond, Maria Varela, Juan Miguel Pascale

**Affiliations:** 1 Departmento de Genómica y Proteómica, Instituto Conmemorativo Gorgas de Estudios de la Salud, Calle 35, Ave Justo Arosemena, Panamá, Panamá; 2 William Paterson University, 300 Pompton Rd, Wayne, New Jersey, 07470, United States of America; 3 St. Michael’s Medical Center, 111 Central Avenue, Newark, New Jersey, 07102, United States of America; 4 Facultad de Medicina, Universidad de Panamá, Transístmica, Panamá, Panamá; 5 Hospital Santo Tomás, Calle 37 Este, Panamá, Panamá; Rutgers University, UNITED STATES

## Abstract

**Background:**

Sexually transmitted infections (STIs) are common in adolescents worldwide. Vulnerability to STIs increases with risky sexual practices. This study described the sexual practices, estimated the prevalence of STIs, and identified correlates associated with STIs among participants, enrolled in public high schools, in the District of Panama, Panama.

**Methods:**

A cross sectional study, using multistage cluster sampling, was conducted among participants, aged 14–18 years, enrolled in public high schools, in the District of Panama, Panama City, Panama, from August to November, 2015. Participants completed a self-administered questionnaire and provided biological samples. The samples of those reporting sexual activity (oral, vaginal, and/or anal intercourse) were tested for STIs. Odds ratios were used to identify correlates of STIs in this population.

**Results:**

A total of 592 participants were included, of whom, 60.8% reported a history of sexual activity, and 24.4% tested positive for least one STI. STIs were more common in female participants, (33.5%). Compared to those without STIs, higher proportions of those with at least one STI reported ≥3 sexual partners in their lifetime (60.0%) and current sexual activity (76.3%). In the multivariable model, correlates of STI included female participants (Adjusted Odds Ratio (AOR) = 5.8, 95% Confidence Interval (CI) 2.3–14.6) and those who engaged in sexual intercourse with casual partners (AOR = 3.0, 95% CI: 1.2–7.5).

**Conclusions:**

We report a high STI prevalence among adolescents attending public high schools, in the District of Panama. Reported risky sexual practices were common and correlated with STIs. Female participants and those reporting sexual intercourse with casual partners were more likely test positive for at least one STI. Our study identified a need for effective interventions to curb future infections in this population.

## Introduction

Adolescents are at an increased risk for sexually transmitted infections (STIs) compared to other age groups. During middle to late adolescence (14–19 years), many individuals engage in their first sexual experience and in sexual practices that may lead to acute infections and conditions that last into adulthood [1–3]. Risky sexual practices such as early sexual initiation, multiple sexual partners, and inconsistent condom use, are common during adolescence [1, 4–6]. The 2009 National Survey of Sexual and Reproductive Health (ENASSER) in Panama found that sexual debut before the age of 18 was common in both sexes (females [50%], males [66%]) [7]. Additionally, adolescent males (15–19 years old) were more likely to have two or more sexual partners than females in the year prior to the survey (38.2% and 21.9%, respectively).

In Panama, the prevalence of HIV in adults (≥15 years), in 2015, was 0.7% [8]. Those aged 15–19 years accounted for 6.8% of all new diagnoses from 2010–2015 [9, 10]. Other STIs are diagnosed syndromically [11]. Reports of STI to Panama’s national surveillance system, in 2014, among 15–19 year olds, included 228 cases of *Neisseria gonorrhoeae*, 10 cases of *Trichomonas vaginalis*, 175 cases of pelvic inflammatory disease, and 0 cases of *Chlamydia trachomatis* (CT) [12]. There are no previous studies describing the prevalence of STIs among adolescents in Panama. However, a community-based study in Mexico reported chlamydia was the most common STI; prevalence in 15–18 year old males and females was 7% and 10%, respectively [13]. We expect a reported history of sexual activity similar to ENASSER and STI prevalence to be between 7% and 10%, for male and female participants, respectively [7, 13]. Increased knowledge of STI prevalence, demographic data and sexual practices related to these infections among high school students may lead to the creation or improvement of programs and policies for the prevention of these infections.

This study will describe the reported sexual practices and determine the prevalence of STIs among participants, aged 14–18 years, enrolled in public high schools (10^th^-12^th^ grades), in the District of Panama. Furthermore, we aim to identify demographic and sexual practice correlates of STIs in this population.

## Methods

### Procedure

The Comité de Bioética del Instituto Conmemorativo Gorgas de Estudios de la Salud reviewed and approved the study protocol. We obtained approval from the Ministry of Education and school administration to conduct the study in selected schools. Underage students (14–17 years old) were given letters to take to their parent/guardian, inviting them to meet with the study team and sign an informed consent. Students of legal age (18 years old) signed their own consent forms. To ensure confidentiality, participant´s names were not used. Participants were assigned unique codes used to identify the questionnaire, samples and results. Additionally, all participants could sign their assent or consent with a real or fictitious name. Neither participants nor guardians were offered monetary compensation. As an incentive, participants were offered a flash memory drive when they returned for their results; additionally they were offered male condoms and personal lubricant.

### Sample

Self-weighting, multistage cluster sampling was used in this cross sectional study. Simple random sampling was used to select ten high schools (from a total of 26). Simple random sampling was again used to select 110 classrooms (grades 10–12) from the 10 schools. Participants in this study included those who completed the consent/assent procedure. We calculated the sample size based on 95% power for each cluster, and on the expected prevalence of *C*. *trachomatis* infection of 10%, similar to a previous study [13].

In 2015, there were 21,702 adolescents, matriculated in 10^th^-12^th^ grades in public schools in the District of Panama [14]. The inclusion criteria for this study were: to be matriculated in 1) a selected public high school, 2) a selected classroom and 3) to be between 14–18 years old.

Between August and November of 2015, 592 male and female participants were included in the study. Guardians of 383 students, aged 14–17 years, signed the informed consent. All minors present on the day the study team visited the school (n = 362) agreed to participate and signed an informed assent form. After agreeing to participate, those of legal age (n = 230) signed an informed consent form. Two guardians declined participation for their female minor student, indicating that she was not sexually active, and five 18-year-old students declined to participate, all indicating they were too busy with schoolwork. Students were not included if absent from school on the day of sampling (21 underage participants).

### Demographic and Sexual Practices Questionnaire

The questionnaire was developed and adapted from previously used national and international instruments [15–17]. Prior to its use, the format and content were validated in an urban high school not selected for inclusion in the study. The questionnaire was self-administered by participants on tablet computers using EpiInfo™ Companion for Android (CDC, Atlanta, Georgia, USA).

#### Socio-demographic Variables

Data collected on socio-demographic variables included: 1) sex, 2) age (in years), 3) school grade, 4) district of residence, 5) religion (Catholicism, Non-Catholic Christianity, and other [any other religion and no religion]), 6) Who do you live with? (two parents [two biological or one biological and one step-parent], other [living with a single parent, extended family, alone, an employer or a romantic partner]).

#### Sexual Practices

All participants were asked eight questions structured differently and spaced throughout the questionnaire about a previous sexual encounter. These questions were: 1) history of sexual activity (yes/no), 2) vaginal sex (yes/no), 3) anal sex (yes/no), 4) oral sex (yes/no), 5) condom use (never engaged in sexual activity, consistent [throughout the whole sexual act]/inconsistent [never, sometimes, or part of the sexual act]), 6) Forced sex by someone they know or do not know (yes/no), 7) Forced sex by boyfriend or girlfriend (yes/no), 8) Have you ever sold sex in exchange for money/food/housing (yes/no)? If at least one of these questions was answered affirmatively then the student was considered to have a past sexual history. Additional questions regarding sexual practices were asked if they indicated a past sexual history. Variables on reported sexual history and practices included: 1) age at sexual debut, 2) first person with whom they had sexual intercourse (boyfriend/girlfriend/spouse, other person [family member, babysitter, neighbor]), this last category from the ´other´ section was included due to national interest in the prevalence of these encounters. Additional variables included: 3) reported age difference between participant and first sexual partner (younger, same age, older [1–9 years, ≥10 years]), 4) reported sexual intercourse with casual partners (ever engaged in sexual activity with someone who was not your boyfriend or girlfriend [yes/no]), 5) number of reported lifetime sex partners (1, 2 and ≥3), cutoff at 3 lifetime partners due to smaller bins at ≥4. Other variables included: 6) current sexual activity (reported intercourse in the past month [yes/no]), 7) previous possible STI (yes/no [belief of having been infected with an STI]), and 8) previous STI diagnosis by a medical professional (yes/no [chlamydia, gonorrhea, HPV or genital warts, HIV, syphilis, hepatitis B]).

### Biological Data and Laboratory Methods

All participants gave blood and urine samples. A trained phlebotomist drew 8 milliliters of blood by venipuncture; urine samples were self-collected by participants. Samples were transported from each high school to the Gorgas Memorial Institute laboratory at controlled temperatures (21-23°C for blood samples and 4°C for urine samples). Although all participants were asked to give samples, only those individuals who indicated a past sexual history were tested for HIV and other STIs. All participants were offered a complete blood count and urinalysis (results not reported here). Blood samples were used for HIV testing with a 4^th^ generation rapid test (Determine™ HIV-1/2 Ag/Ab, Alere Medical Co. Ltd., Japan/Israel). Positive rapid tests were confirmed by viral load testing (RealTi*m*e HIV-1, Abbot Molecular Inc., USA). Urine samples were evaluated for STIs: *Neisseria gonorrhoeae*, *Chlamydia trachomatis*, *Mycoplasma genitalium* and *Trichomonas vaginalis* (N.gonorrhoeae/С.trachomatis/M.genitalium/T.vaginalis Real-TM, Sacace™, Italy). To lower the chance of RT-PCR false positive results, if 3 consecutive positive results occurred in a run, the testing was repeated with a different RT-PCR well-placement order. Samples that were positive in both runs were reported as positive. Due to warm weather some urine samples degraded during transport and were not included in the analyses (n = 23).

On the day of sampling, participants were given an appointment, in two weeks, to obtain their results from the health center closest to their high school. The majority (60.2%) of STI positive participants returned for their test results and treatment. Those who kept their appointments received post-test counseling, linkage to care and treatment from a team of trained physicians, psychologists and social workers (based on national guidelines [11, 18]). They were offered mental health counseling and were referred for further care, if needed. Those with a HIV positive result were accompanied for follow-up care and treatment with the Ministry of Health.

### Data Analyses

Questionnaires were uploaded from tablet computers to a database using Epi Info V7.1.5.0, (CDC, Atlanta, Georgia, USA). Participants who completed the questionnaire and whose biological samples were tested for STIs were included in the following analyses.

Univariable, bivariable and regression analyses were conducted using Stata V12.0 (StataCorp, College Station, Texas, USA). Socio-demographic characteristics of participants were compared by sexual activity. The prevalence of HIV, CT, *Neisseria gonorrhoeae*, *Trichomonas vaginalis* and *Mycoplasma genitalium* was stratified by gender. We used *χ*^2^ to evaluate the association between a history of sexual activity and socio-demographic variables; gender and sexual practices variables and the relationship between STI and socio-demographic or sexual practices variables. We used Fisher’s exact test to evaluate the association between STIs and gender due to small bin sizes. Kruskal- Wallis Test for trend was used to evaluate trends in age, grade, and number of casual partners and STIs. Missing data were not analyzed. Statistical significance was set at p<0.05. Variables significant at p<0.2 were included in the regression model. The odds ratio (OR) was used to evaluate correlations between socio-demographic variables, sexual practices and STI.

## Results

### Reported Socio-demographic Variables and Sexual Activity

A total of 592 participants were included in this study. The majority lived in the District of Panama, 475/592 (80.2%) and 356/592 (60.1%) were female (Table 1). The mean age for participants was 16.9 years (SD = 1.1); (females 16.9 years, SD = 1.1; males 17.0 years, SD = 1.0). A history of sexual activity was reported by 360/592 participants (60.8%) and was more likely among older participants and those in higher grades (Kruskal-Wallis Test for trend, p<0.005) (Table 1).

**Table 1:** Reported Characteristics of Participants, by History of Sexual Activity, Panama, 2015.

		All participants		History of Sexual Activity				
				No		Yes		p-value
		n	%	n	%	n	%	
Gender		592		212		360		0.365
	**Female**	356	60.1	122	57.5	221	61.4	
	**Male**	236	39.9	90	42.5	139	38.6	
Age (in years)		592		211		360		**<0.001**^**!**^
	**14–15**	84	14.2	39	33.6	36	10.0	
	**16**	133	22.5	71	24.6	59	16.4	
	**17**	144	24.4	49	23.2	87	24.2	
	**18**	230	38.9	39	18.5	178	49.4	
School grade		591		212		359		**<0.001**^**!**^
	**10th**	166	28.1	81	38.2	75	20.9	
	**11th**	159	26.9	69	32.6	84	23.4	
	**12th**	266	45.0	62	29.2	200	55.7	
District of residence		592		212		360		0.481
	**Panama**	475	80.2	173	81.6	285	79.2	
	**Other**	117	19.8	39	18.4	75	20.8	
Religion		235		88		140		0.623
	**Catholic**	118	50.2	47	53.4	67	47.9	
	**Christian (non-Catholic)**	85	36.2	29	33.0	55	39.3	
	**Other**^a^	32	13.6	12	13.6	18	12.9	
Who do you live with?		573		207		349		**0.009**
	**2 parents**^b^	383	66.8	151	72.9	217	62.2	
	**Other**^c^	190	33.2	56	27.1	132	37.8	

*χ*^2^ was used to evaluate the association between a history of sexual activity and included variables.

^!^Kruskal-Wallis Test for trend was statistically significant at p<0.05

^a^“Other” refers to any other religion or “no religion”.

^b^Two biological parents or one biological and one step-parent.

^c^One parent, another family member, lives with romantic partner or alone.

Note: numbers differ in each category differ due to number of responses.

### Reported Sexual Practices

The majority of participants with a history of sexual activity reported current sexual activity, 198/336 (58.9%), with a higher proportion of females than males reporting current sexual activity, 133/203 (65.6%) and 65/133 (48.9%), respectively (p = 0.002) (Table 2). Female participants were more likely to have their sexual debut with someone 1–9 years older, (110/172, 63.9%; p<0.001). Male participants were more likely to engage in sexual intercourse with a casual partner (73/134, 54.5%; p = 0.001) and have three or more partners in their lifetime (62/102, 60.8%; p = 0.003). At the time of the study, very few participants, 15/365 (4.1%) reported a previous STI diagnosis and no participants indicated a positive HIV test (Table 2).

**Table 2:** Reported Sexual Practices Among Participants with a History of Sexual Activity, Panama, 2015.

		Total		Females		Males		
		n	%	n	%	n	%	p-value
Vaginal sex		305		195		110		**0.001**
	Yes	258	84.6	175	89.7	83	75.5	
Anal sex		308		192		116		0.315
	Yes	88	28.6	51	26.6	37	31.9	
Oral sex		307		192		115		0.353
	Yes	147	47.9	88	45.8	59	51.3	
Age of sexual debut		259		170		89		0.082
	≤14 years	81	31.3	47	27.6	34	38.2	
	≥15 years	178	68.7	123	72.4	55	61.8	
Relation with first sexual partner		287		181		106		**<0.001**
	Friend or classmate	59	20.6	23	12.7	36	34.0	
	Boyfriend, girlfriend or spouse	206	71.8	146	80.7	60	56.6	
	Other^a^	22	7.7	12	6.6	10	9.4	
Age difference with first sexual partner		280		172		108		**<0.001**
	Older than me (10 years or more)	25	8.9	15	8.7	10	9.3	
	Older than me (1–9 years)	149	53.2	110	63.9	39	36.1	
	The same age or younger than	106	37.9	47	27.3	59	54.6	
Condom use (consistent)		302		188		114		0.961
	Yes	79	26.2	49	26.1	30	25.3	
Sex with casual partner		345		211		134		**0.001**
	Yes	150	43.5	77	36.5	73	54.5	
Number of sex partners in lifetime		272		170		102		**0.003**
	1	81	29.8	59	34.7	22	21.6	
	2	62	22.8	44	25.9	18	17.6	
	≥3	129	47.4	67	39.4	62	60.8	
Current sexual activity^b^		336		203		133		**0.002**
	Yes	198	58.9	133	65.6	65	48.9	
Sell sex in exchange for money/food/ housing		330		205		125		0.751
	Yes	25	4.6	16	4.8	9	4.3	
Previous possible STI or HIV infection^c^		335		188		133		0.907
	Yes	125	38.6	73	37.1	52	37.8	
Previous STI diagnosis^d^		365						0.514
	Yes	15	4.1	8	3.6	7	5.0	

*χ*^2^ was used to evaluate sexual activity between males and females.

^**a**^Family member (cousin, aunt, uncle, any other family member), babysitter or neighbor.

^**b**^At least one sexual partner in the past month.

^**c**^Thought to have been infected by a sexual partner.

^**d**^Been previously diagnosis by a doctor.

Note: Different numbers in each category based on the number of responses

### HIV and Other STI Results

Overall, 88/360 (24.4%) of participants with a reported history of sexual activity tested positive for at least one STI. *Chlamydia trachomatis* was found in 72/337 (21.4%) of participants, with a higher proportion of females than males testing positive, 64/207 (30.9%) and 8/130 (6.2%), respectively (p<0.001). Only male participants 3/139 (2.2%) tested positive for HIV (Table 3). All individuals who tested positive for *Neisseria gonorrhoeae* also tested positive for CT (n = 6).

**Table 3:** Prevalence of Sexually Transmitted Infections Among Participants with a History of Sexual Activity, by Sex, Panama, 2015.

	Total			Females			Males			
	Total tested^a^	Positive	%	Total tested	Positive	%	Total tested	Positive	%	p-value
**HIV^b^**	360	3	0.8	221	0	-	139	3	2.2	0.057
***Chlamydia trachomatis***^c^	337	72	21.4	207	64	30.9	130	8	6.2	**<0.001**
***Neisseria gonorrhea***^c^	337	6	1.8	207	6	2.9	130	0	0.0	0.086
***Mycoplasma genitalium***^c^	337	15	4.5	207	12	5.8	130	3	2.3	0.177
***Trichomonas vaginalis***^c^	337	6	1.8	207	6	2.9	130	0	0.0	0.086
**At least 1 infection**^d^	360	88	24.4	221	74	33.5	139	14	10.1	**<0.001**

Fisher’s exact was used to evaluate the prevalence of STI between males and females as there were cell sizes <5

^**a**^Laboratory tests were performed only on participants that indicated history of sexual activity.

^**b**^4th generation HIV Rapid Test, confirmed by viral load.

^**c**^Real-time polymerase chain reaction.

^**d**^ One or more of: HIV, *C*. *trachomatis*, *N*. *gonorrhea*, *M*. *genitalium*, *T*. *vaginalis*.

Note: Some urine samples degraded in transport to the lab, thereby explaining the number of STI tests performed.

### Correlates of STI

A higher proportion of female participants tested positive for at least one STI, compared to males, 74/221 (33.5%) and 14/139 (10.1%; Tables 3 and 4). Older participants and those in a higher school grade accounted for a higher proportion of observed STIs (Kruskal-Wallis Test p<0.05). The majority of those with a STI reported risky sexual practices: inconsistent condom use, 66/80 (82.5%), sexual intercourse with casual partner(s), 51/80 (63.8%), and 3 or more lifetime sex partners 45/75 (60.0%). The majority of the participants 61/80 (76.3%) with a STI reported being sexually active within the month prior to sampling (Table 4).

**Table 4:** Correlates of STIs Among Participants (14–18 years old), Panama, 2015.

		Sexually Transmitted Infection						
		Positive		Negative				
		n	%	n	%	p-value	OR (95%CI)	AOR (95%CI)
Gender		88		272		**<0.001**		
	**Female**	74	84.1	147	54.0		4.5 (2.4–8.3)	5.8 (2.3–14.6)
	**Male**	14	15.9	125	46.0		1.0	1.0
Age (in years)		88		272		**0.029**^!^		
	**14–15**	7	8.0	29	10.7		1.0	1.0
	**16**	7	8.0	52	19.1		0.6 (0.2–1.7)	0.2 (0.1–3.3)
	**17**	20	22.7	67	24.6		1.2 (0.5–3.2)	0.3 (0.1–4.7)
	**18**	54	61.4	124	45.6		1.8 (0.7–4.4)	0.3 (0.1–5.0)
School grade		87		272		**0.037**^!^		
	**10th**	11	12.6	64	23.5		1.0	1.0
	**11th**	18	20.7	66	24.3		1.6 (0.7–3.6)	4.7 (0.5–48.1)
	**12th**	58	66.7	142	52.2		2.4 (1.2–4.8)	4.7 (0.4–52.6)
Reported vaginal sex		79		226		**0.009**		
	**Yes**	74	93.7	184	81.4		3.4 (1.2–8.9)	1.2 (0.3–4.6)
Reported oral sex		75		232		0.105		
	**Yes**	42	56.0	105	45.3		1.5 (0.9–2.6)	0.7 (0.3–1.4)
Reported age difference with the first sexual partner		67		212		0.152		
	**Older 10 years or more**	8	11.9	17	8.0		1.0	1.0
	**Older 1–9 years**	40	59.7	108	50.9		0.8 (0.3–2.0)	0.9 (0.3–3.4)
	**The same age or younger**	19	28.4	87	41.0		0.5 (0.2–1.2)	0.9 (0.2–3.6)
Reported condom use		80		222		**0.040**		
	**Inconsistent**	66	82.5	157	70.7		1.0	1.0
	**Consistent**	14	17.5	65	29.3		0.5 (0.3–1.0)	1.0 (0.3–2.8)
Reported sex with casual partner		80		229		**0.002**		
	**Yes**	51	63.8	99	43.2		2.3 (1.4–3.9)	**3.0 (1.2–7.5)**
Reported number of sex partners in lifetime		75		197		**0.006**^!^		
	**1**	12	16.0	69	35.0		1.0	1.0
	**2**	18	24.0	44	22.3		2.4 (1.0–5.4)	1.4 (0.5–4.6)
	**≥3**	45	60.0	84	42.6		3.1 (1.5–6.3)	1.9 (0.6–5.9)
Reported current sexual activity^a^		80		227		**0.005**		
	**Yes**	61	76.3	133	58.6		2.3 (1.3–4.1)	1.4 (0.6–3.4)
Belief of STI or HIV at some time^b^		78		243		0.117		
	**Yes**	36	46.2	88	36.2		1.5 (0.9–2.5)	1.2 (0.6–2.8)

Abbreviations: OR = Odds Ratio, AOR = Adjusted Odds Ratio, CI = Confidence Interval, STI = Sexually Transmitted Infection, HIV = Human Immunodeficiency Virus

*χ*^2^ was used to calculate the relationship between sexually transmitted infections and included variables.

^!^ Kruskal-Wallis Test for trend was statistically significant at < 0.05.

^a^One or more sexual partner in the past month,.

^b^Thought to have been infected by a sexual partner.

Note: Different numbers in each category based on the number of responses.

### Multivariable Analyses

In the adjusted model, female participants (Adjusted Odds Ratio (AOR = 5.8, 95% Confidence Interval (CI) 2.3–14.6) and those who reported sexual intercourse with casual partners (AOR = 3.0, 95% CI 1.2–7.5) were found to be more likely to have a STI (Table 4).

## Discussion

This cross sectional study was conducted in male and female, 10^th^-12^th^ grade high school students, in the District of Panama, Panama. We found a high proportion of 14–18 years old participants reporting a history of sexual activity, many of whom reported risky sexual practices and sexual relations with three or more total lifetime partners. Almost half had engaged in sexual intercourse with casual partners, and few consistently used condoms. These results are consistent with what has been found regionally in Panama and Mexico [7, 13]. Risky practices have been shown to put adolescents at greater risk for infection [4–6, 19, 20]. Practices such as early sexual debut and increased numbers of sexual partners have been found to be more common in adolescent males than females [7, 21, 22]. We found that a large proportion of adolescent females reported their first sexual experience with an older partner. Adolescent females often engage in age-discordant sexual relationships, putting themselves at risk for sexual and reproductive health outcomes [23, 24]. We found that participants had a higher STI burden than what was previously seen in a community-based study [13]. Programs and policies to prevent further STIs and risky sexual may prevent an even higher burden of disease among adolescents.

Genital *Chlamydia trachomatis*, the most common bacterial STI in the US, is most prevalent in the adolescent and young adult population [25, 26]. Over 20% of sexually active participants had a positive CT test, a prevalence much higher than expected, and higher than regional studies have found in similar populations [13, 25, 27]. The majority of participants with a STI in our study indicated they had not previously sought treatment for a STI, suggesting that these infections were mostly asymptomatic. Asymptomatic CT infection was previously noted to be common, especially in young females [26, 28, 29]. Although CT is classified as a reportable disease in Panama, annual reports in this age group show zero cases; other STIs are also poorly documented [12, 30, 31]. Only female participants tested positive for either *Trichomonas vaginalis* or *Neisseria gonorrhoeae*; a number of female participants tested positive for *Mycoplasma genitalium*. Therefore, the potential for onward transmission of these microorganisms remains. Additionally, the presence of CT, *T*. *vaginalis*, *N*. *gonorrhoeae and M*. *genitalium*, may lead to long-term sequelae such as pelvic inflammatory disease, ectopic pregnancy and infertility, thereby possibly affecting the reproductive health of young adults [32–34]. Our study indicates that underreporting of STIs, to the Ministry of Health’s surveillence unit, is most likely occuring for all STIs in this population.

More than 2% of sexually active males tested positive for HIV. This is a surprising result since national surveillance reports that 15–19 year old female adolescents have a higher infection rate than males [10]. At this time, pregnant women participate in routine opt-out HIV screening [9, 10, 35]. Pregnant adolescents may not attend school, and were not included in our sampling. Access to diagnostic STI testing is difficult for adolescents in Panama. According to national guidelines, diagnosis and treatment of STIs are based on clinical presentation and screening and diagnostic testing are not performed; therefore many asymptomatic cases are likely not identified and reported [11]. Although syndromic management is a common practice worldwide, it is especially problematic for those with asymptomatic infections [36, 37]. To additionally complicate STI diagnoses in adolescents in Panama, access to healthcare services, including sexual and reproductive care is difficult, if not impossible, for underage individuals without the presence of their guardian [38].

Prevention strategies including increased screening for STIs among male and female adolescents may identify these infections at an earlier time. This was demonstrated to be cost effective in populations where CT prevalence is >3% [39]. Additionally, as most participants report a history of sexual activity and are sexually active, educational strategies supporting abstinence-only education may not be effective [40–42]. Instead these strategies should emphasize risk reduction interventions, such as lowering the number of sexual partners and increasing condom use [43, 44].

We found that STIs were positively correlated with reported risky sexual practices, as adolescents’ age increased, and with higher school grade. These reported risky practices include: current sexual activity, three or more total lifetime sexual partners and inconsistent condom use, all of which are associated with increased risk of STIs [2, 37, 45]. Correlates of STIs we identified included being a female participant and reported sexual intercourse with a casual partner, similar to worldwide reports [20, 26, 29].

Our study had a number of limitations. In first place, STI prevalence may be underestimated. Adolescents who are at the highest risk for STIs may not attend high school, or their parents did not attend scheduled meetings to sign the consent forms. Lack of parental involvement in school meetings is a common issue in public high schools across the District [46]. Additionally, STI testing was only performed if the student indicated in the questionnaire that they had a history of sexual activity. Secondly, some urine samples were unusable due to degradation of the genetic material caused by the warm climate. Thirdly, there was a disproportionate number of 12^th^ grade classrooms included that may have biased the results, since these participants were more likely to have a history of sexual activity and report a higher number of sexual partners. Fourthly, our study was only conducted in public schools; students attending private schools and those not attending school were not represented. Finally, since this study was carried out in the capital city, in the District of Panama, results may not be representative of all students in public schools across the Republic of Panama.

Despite these limitations, we were able to include a significant number of underage participants, with almost all guardians who attended meetings supporting participation of their minors in the study. Questionnaires were successfully filled out by the participants themselves, which may have decreased response bias.

Our results indicate a very high STI prevalence in the population studied. The majority of the participants reported being sexually active and many reported engaging in risky sexual practices. Female students, and those engaging in sexual intercourse with casual partners, are at increased risk for STIs. Prevention programs that focus on reducing the number of new infections, as well as STI-related morbidity, should be considered for public high school students in the District of Panama.
